# Author Correction: TNFα increases tyrosine hydroxylase expression in human monocytes

**DOI:** 10.1038/s41531-021-00212-8

**Published:** 2021-08-02

**Authors:** Adithya Gopinath, Martin Badov, Madison Francis, Gerry Shaw, Anthony Collins, Douglas R. Miller, Carissa A. Hansen, Phillip Mackie, Malú Gámez Tansey, Abeer Dagra, Irina Madorsky, Adolfo Ramirez-Zamora, Michael S. Okun, Wolfgang J. Streit, Habibeh Khoshbouei

**Affiliations:** 1 Department of Neuroscience, University of Florida, Center for Translational Research in Neurodegenerative Disease, Norman Fixel Institute for Neurological Diseases, Gainesville, FL USA; 2 Encor Biotechnology Inc., Gainesville, FL USA; 3 Department of Neurology, University of Florida, Gainesville, FL USA

**Keywords:** Neuroimmunology, Enzymes, Parkinson's disease

Correction to: *npj Parkinson’s Disease* 10.1038/s41531-021-00201-x, published online 20 July 2021

The original version of the published Article had a mistake in the Competing Interests section. The Competing Interests have been updated to the following: Malú Gámez Tansey is a co-inventor on the XPro1595 patent, and is a consultant to and has stock ownership in INmune Bio, which has licensed XPro1595 for neurological indications. The remaining authors declare no competing interests. The HTML and PDF versions of the Article have been corrected.

