# Supplementary material for: TNFα increases tyrosine hydroxylase expression in human monocytes
Source: NPJ Parkinsons Dis. 2021 Jul 20;7:62. doi: 10.1038/s41531-021-00201-x (PMC8292430; doi:10.1038/s41531-021-00201-x)
Supplement: Supplementary file 1 — Supplementary Information [file 41531_2021_201_MOESM1_ESM.pdf]

Supplementary Figure 1

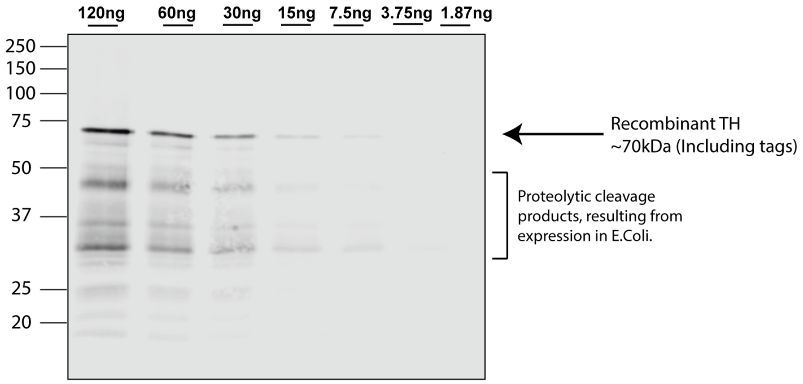

Supplementary Figure 1. Lower molecular weight bands in recombinant TH protein are proteolytic cleavage products resulting from prokaryotic expression of TH protein. Similar to lower molecular weight bands seen in Figure 2, proteolytic cleavage is evident in recombinant TH protein assayed by western blot.

Supplementary Figure 2

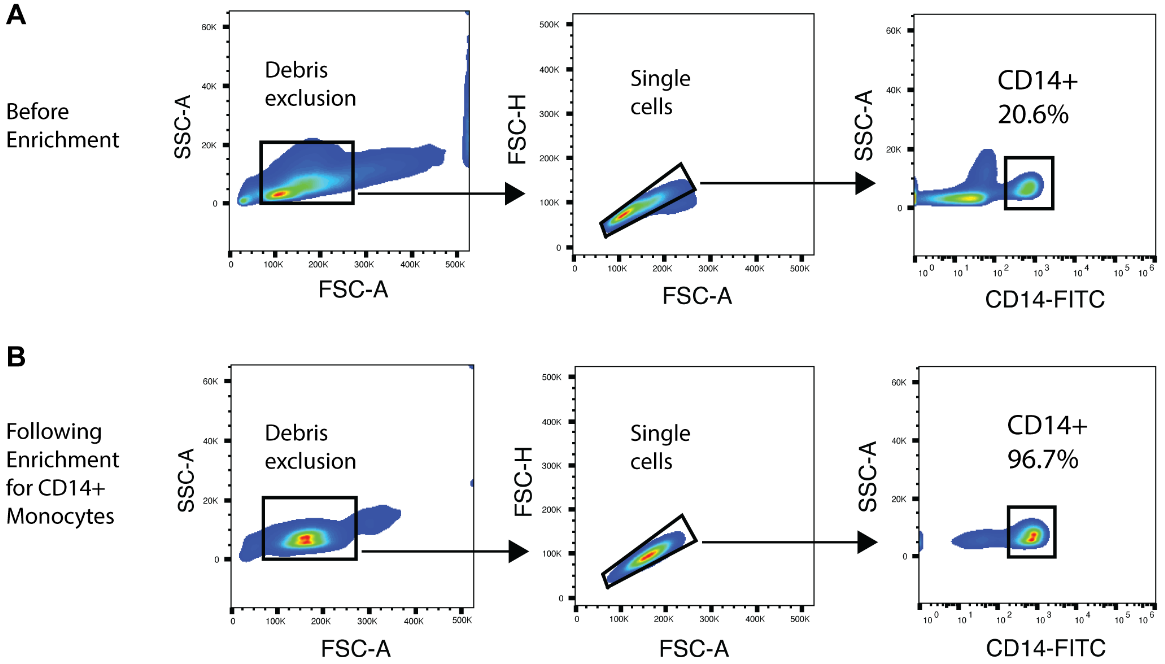

Supplementary Figure 2. Enrichment for CD14+ monocytes from total PBMCs. A) Total PBMCs isolated from healthy donor whole blood shows ~20% CD14+ monocytes as a fraction of total PBMCs. B) Following magnetic selection (see methods), a highly enriched population of monocytes consisting of greater than 95% CD14+ monocytes is available for downstream cell culture treatments.

Supplementary Table 1

Supplementary Table 1: Clinical data for Parkinson's disease patients								
Patient ID	Disease Duration (Years)	Sex	Age (Years)	H-Y	UPDRS Off	UPDRS On	Other Conditions	Medications
Parkinson's 149	7	M	71	2	33	--	None	Rytary, Ropinirole, Amantadine, Azilect, Apokyn
Parkinson's 150	9	M	50	2	--	26	None	Sinemet, Selegiline, Mirapex
Parkinson's 151	19	F	70	2	38	37	RLS	Sinemet, Pramipexole
Parkinson's 154	15	M	76	--	--	--	None	Asprin, Sinemet, Klonopin, Rasagilline, Rigotine
Parkinson's 155	9	M	85	3	--	25	HTN, T2D	Atorvastatin, Sinemet, Zetia, Losrtan, Metformin, Metoprolol, Flomax
Parkinson's 161	2	M	61	2	--	33	HTN	Amantadine, Sinemet, Atorvastatin, Losartan, Metoprolol, Omeprazol
Parkinson's 163	7	M	70	3	15	--	None	Sinemet, Amantadine, Flomax, Ropinirole
Parkinson's 164	4	F	67	--	17	--	None	Atorvastatin, Sinemet, Fluoxetine, Ganapentin, Losartan
Parkinson's 165	1	M	60	3	--	23	Arthritis	Sinemet, Amantadine, Celebrex, Sertraline, Trazadone
Parkinson's 166	3	F	67	3	--	--	None	Sinemet, Tylenol
Parkinson's 167	6	M	59	2	--	26	T2D	Sinemet, Asprin, Amlodipine, Metformin, Ibuprofen
Abbreviations: RLS (restless leg syndrome), HTN (hypertension), T2D (type 2 diabetes), H-Y (Hoen Yar), UPDRS (universal Parkinson's disease rating scale)								

Supplementary Table 1: Clinical data for Parkinson's disease patients. All PD patients and healthy control subjects were free from blood borne pathogens, viral/bacterial infections, had not been treated for infections within the preceding 21 days and were not taking blood thinners other than aspirin. Detailed medical histories for healthy control subjects were not available, other than those data presented. UPDRS Off represents Part 3 motor scores when subjects were not currently administered dopamine replenishment therapy (L-DOPA/Sinemet). UPDRS On represents Part 3 motor scores 30 minutes following dopamine replenishment therapy (L-DOPA/Sinemet).
