# Supplementary material for: TNFα increases tyrosine hydroxylase expression in human monocytes
Source: NPJ Parkinsons Dis. 2021 Jul 20;7:62. doi: 10.1038/s41531-021-00201-x (PMC8292430; doi:10.1038/s41531-021-00201-x)
Supplement: Supplementary file 2 — Reporting Summary [file 41531_2021_201_MOESM2_ESM.pdf]

nature research

Corresponding author(s): Adithya Gopinath

Last updated by author(s): 5/15/2021

Reporting Summary

Nature Research wishes to improve the reproducibility of the work that we publish. This form provides structure for consistency and transparency in reporting. For further information on Nature Research policies, see our Editorial Policies and the Editorial Policy Checklist.

Statistics

For all statistical analyses, confirm that the following items are present in the figure legend, table legend, main text, or Methods section.

n/a	Confirmed	
☐	☒	The exact sample size (n) for each experimental group/condition, given as a discrete number and unit of measurement
☐	☒	A statement on whether measurements were taken from distinct samples or whether the same sample was measured repeatedly
☐	☒	The statistical test(s) used AND whether they are one- or two-sided<br>*Only common tests should be described solely by name; describe more complex techniques in the Methods section.*
☒	☐	A description of all covariates tested
☐	☒	A description of any assumptions or corrections, such as tests of normality and adjustment for multiple comparisons
☐	☒	A full description of the statistical parameters including central tendency (e.g. means) or other basic estimates (e.g. regression coefficient) AND variation (e.g. standard deviation) or associated estimates of uncertainty (e.g. confidence intervals)
☐	☒	For null hypothesis testing, the test statistic (e.g. F, t, r) with confidence intervals, effect sizes, degrees of freedom and P value noted<br>*Give P values as exact values whenever suitable.*
☒	☐	For Bayesian analysis, information on the choice of priors and Markov chain Monte Carlo settings
☒	☐	For hierarchical and complex designs, identification of the appropriate level for tests and full reporting of outcomes
☒	☐	Estimates of effect sizes (e.g. Cohen's d, Pearson's r), indicating how they were calculated

Our web collection on statistics for biologists contains articles on many of the points above.

Software and code

Policy information about availability of computer code

Data collection	BD FACSDiva and Sony Flow Cytometry software (Flow cytometry)
Data analysis	FlowJo (Flow Cytometry) and Graphpad Prism (Statistics and analysis)

For manuscripts utilizing custom algorithms or software that are central to the research but not yet described in published literature, software must be made available to editors and reviewers. We strongly encourage code deposition in a community repository (e.g. GitHub). See the Nature Research guidelines for submitting code & software for further information.

Data

Policy information about availability of data

All manuscripts must include a data availability statement. This statement should provide the following information, where applicable:

- Accession codes, unique identifiers, or web links for publicly available datasets
- A list of figures that have associated raw data
- A description of any restrictions on data availability

Data is available upon reasonable request.

Field-specific reporting

Please select the one below that is the best fit for your research. If you are not sure, read the appropriate sections before making your selection.

☒ Life sciences ☐ Behavioural & social sciences ☐ Ecological, evolutionary & environmental sciences

For a reference copy of the document with all sections, see nature.com/documents/nr-reporting-summary-flat.pdf

Life sciences study design

All studies must disclose on these points even when the disclosure is negative.

Sample size	Sample sizes were not predetermined, and instead followed previous publications from our lab and others.
Data exclusions	No data was excluded.
Replication	All data were generated from multiple biological replicates
Randomization	All samples were assigned treatments randomly.
Blinding	All experiments and analysis were performed blindly.

Reporting for specific materials, systems and methods

We require information from authors about some types of materials, experimental systems and methods used in many studies. Here, indicate whether each material, system or method listed is relevant to your study. If you are not sure if a list item applies to your research, read the appropriate section before selecting a response.

Materials & experimental systems

n/a	Involved in the study	
☐	☒	Antibodies
☐	☒	Eukaryotic cell lines
☒	☐	Palaeontology and archaeology
☐	☒	Animals and other organisms
☐	☒	Human research participants
☐	☒	Clinical data
☒	☐	Dual use research of concern

Methods

n/a	Involved in the study	
☒	☐	ChIP-seq
☐	☒	Flow cytometry
☒	☐	MRI-based neuroimaging

Antibodies

Antibodies used	Anti TH (MCA-4H2, RPCA-TH: Encor Biotechnologies); Anti TH (AB152: Millipore-Sigma); anti-Ki67 (CPCA-Ki67: Encor Biotechnologies); Anti HSP60 (CPCA HSP60: Encor Biotechnologies)
Validation	All antibodies were independently validated by the vendors (Encor and Millipore-Sigma websites and product pages). In addition, in this manuscript we conducted independent validation of MCA-4H2 and RPCA-TH.

Eukaryotic cell lines

Policy information about cell lines

Cell line source(s)	In house.
Authentication	None of the cell lines were authenticated.
Mycoplasma contamination	These cell lines were not assayed for mycoplasma contamination
Commonly misidentified lines (See ICLAC register)	N/A

Animals and other organisms

Policy information about studies involving animals; ARRIVE guidelines recommended for reporting animal research

Laboratory animals	Mouse pups of both sexes were used for preparation of primary dopamine neuron cultures.
Wild animals	This study did not involve wild animals
Field-collected samples	This study did not involve field-collected samples.
Ethics oversight	All experiments were approved by the Institutional Animal Care and Use Committee at University of Florida.

Note that full information on the approval of the study protocol must also be provided in the manuscript.

Human research participants

Policy information about studies involving human research participants

Population characteristics	Male and female Parkinson's disease patients and healthy controls were recruited between age 40-80, with no blood borne diseases, pathogens and no specific genetic characteristics. Parkinson's patients were in a wide range of time from diagnoses and disease severity.
Recruitment	Participants were recruited based on diagnoses made by trained neurologists, after obtaining written informed consent. No self-selection biases are known.
Ethics oversight	University of Florida IRB

Note that full information on the approval of the study protocol must also be provided in the manuscript.

Clinical data

Policy information about clinical studies

All manuscripts should comply with the ICMJE guidelines for publication of clinical research and a completed CONSORT checklist must be included with all submissions.

Clinical trial registration	*Provide the trial registration number from ClinicalTrials.gov or an equivalent agency.*
Study protocol	*Note where the full trial protocol can be accessed OR if not available, explain why.*
Data collection	*Describe the settings and locales of data collection, noting the time periods of recruitment and data collection.*
Outcomes	*Describe how you pre-defined primary and secondary outcome measures and how you assessed these measures.*

Flow Cytometry

Plots

Confirm that:

☒ The axis labels state the marker and fluorochrome used (e.g. CD4-FITC).

☒ The axis scales are clearly visible. Include numbers along axes only for bottom left plot of group (a 'group' is an analysis of identical markers).

☒ All plots are contour plots with outliers or pseudocolor plots.

☒ A numerical value for number of cells or percentage (with statistics) is provided.

Methodology

Sample preparation	Whole blood was collected in K2EDTA vacutainer blood collection tubes (BD, 366643) and held at room temperature for up to 2 hours prior to PBMC isolation. Briefly, blood from healthy volunteers and PD patients was overlaid in Leucosep tubes (Table 2) for PBMC isolation, centrifuged for 20 minutes at 400g with brakes turned off and acceleration set to minimum. PBMCs were collected from the interphase of Ficoll and PBS, transferred to a fresh 15mL conical tube, resuspended in 8mL sterile PBS and centrifuged for 10 minutes at 100g, and repeated twice more. Cells were counted with a hemacytometer using trypan blue exclusion of dead cells, and density-adjusted for downstream applications.
Instrument	Sony Spectral Analyzer SP6800
Software	FlowJo (TreeStar Inc)
Cell population abundance	25,000 total events were collected. Debris were excluded, followed by doublet exclusion by FSC-A x FSC-H gating. The final population analyzed contained at least 10,000 cells. A maximum of 3 fluorescent parameters per sample were analyzed.

Depending on the treatment and experimental group, between 3000 and 8000 cells were present in the final analysis gate.

Gating strategy

FSC-A x SSC-A plots were used to exclude debris below 50,000 units. FSC-A x FSC-H plots were used to exclude doublets per established protocols. The final analysis plot was gated on SSC-A x Marker (i.e. CD14+ or TH+). Gates for final analysis were set based on separation from Isotype Control.

☒ Tick this box to confirm that a figure exemplifying the gating strategy is provided in the Supplementary Information.
